# Nipple-Sparing Mastectomy Long-Term Outcomes: Early and Late Complications

**DOI:** 10.3390/medicina56040166

**Published:** 2020-04-08

**Authors:** Alessio Metere, Elisabetta Fabiani, Maria Teresa Lonardo, Domenico Giannotti, Daniela Pace, Laura Giacomelli

**Affiliations:** 1 Surgical Sciences Department, “Sapienza” University of Rome, Viale Regina Elena 261, 00161 Rome, Italy; laura.giacomelli@uniroma1.it; 2 Emergency Department, Aurelia Hospital, Via Aurelia, 860, 00165 Rome, Italy; elisabetta_fabiani@yahoo.it; 3 Department of Surgery, Ospedali Riuniti di Anzio-Nettuno, Via Cupa dei Marmi, 00042 Anzio, Italy; mariateresalonardo@libero.it; 4 Department of Surgery, Ospedale Belcolle, Strada Sammartinese snc, 01100 Viterbo, Italy; dome.giannotti82@libero.it; 5 Valmontone Hospital, Via dei Lecci snc, 00038 Valmontone, Italy; dani.pace@tiscalinet.it

**Keywords:** nipple-sparing mastectomy, breast cancer, nipple-sparing outcome, mastectomy

## Abstract

*Background and Objectives:* The surgical choice treatment of the breast cancer mostly depends on the stage of the disease. In the last years, breast cancer surgery has moved from being destructive to being more respectful of the anatomical and physiological integrity of the gland. The aim of the breast surgery should be finalized to obtain the best aesthetic and functional results, respecting the principles of oncologic radicality. The present study is a retrospective analysis aimed to evaluate the long-term outcomes of a conservative technique like the nipple-sparing mastectomy. *Materials and Methods:* We observed 894 patients with a median age of 47.5 years old, underwent nipple-sparing mastectomy between 2002–2017. The data acquired include population and tumor characteristics, patient reconstructive outcomes, including locoregional, regional, and distant metastases; other variables, among nipple–areola complex necrosis and infection were collected. *Results:* The complications detected were considered as “early” within 1 month later the nipple-sparing mastectomy or “late” after this time. The overall complications rate (early and late) and the overall survival and the relapses detected by this study were comparable with those reported in the literature. In order to identify factors that correlate with complications, either early or later, it has been processed an evaluation of the univariate analysis showing adjuvant chemotherapy as the only predictive factor for late complications, while we encountered no predictors for early complications. *Conclusions:* The present study adds to the data already present in literature, demonstrating that the nipple-sparing mastectomy is a safe procedure, providing good oncological and aesthetic results in patients carefully selected.

## 1. Introduction

Breast cancer was the third most common incident cancer overall with an estimated 2.0 million (95% UI, 1.9–2.0 million) incident cases in 2017. It caused 601,000 (95% UI, 579,000–630,000) deaths in women and 11,000 (95% UI, 10,000–11,000) deaths in men, making it the fifth leading cause of cancer deaths for both sexes combined in 2017 globally and the first for women [1]. Breast cancer is characterized by a wide variety of clinical presentations and degree of aggressiveness. Today the new programs of breast screening allow an early diagnosis. The choice between medical or surgical treatment depends on the stage of the cancer; in most initial cases conservative treatment is allowed.

### 1.1. Breast Cancer Surgical Treatment

Surgical treatment of breast cancer has moved from being aggressive and destructive to being respectful of anatomical and physiological integrity [2]. Aggressive and mutilating surgery, which was conceived in the past, has now become outdated [3]. Enlarged and super-enlarged mastectomy interventions can now be considered part of the history of breast surgery, although they are not anymore presentable as techniques to use, if not for selected cases; even Halsted mastectomy, is indicated only for interventions in conditions of locally advanced disease, for the purposes of neoplastic reductions or for palliative care of local phenomena such us hemorrhage or infection [4]. The choice of the type of surgical intervention should fall on the most conservative technique possible, trying to obtain the best aesthetic and functional results, while respecting the principles of oncologic radicality. The term conservative surgery includes all of the interventions (quadrantectomy/tumorectomy with or without associated axillary lymphadenectomy) that propose to remove the neoplasia with negative surgical margins defined by the absence of any ink on the excised tumor (no ink tumor). This therapeutical approach has been confirmed by various pointed out superimposable survival percentages between patients treated with mastectomy and those treated with conservative surgery [5].

### 1.2. Conservative Mastectomies

Despite nowadays being the era of conservative surgery, demolition surgery of the breast is still indicated today, but more limited than the past. This intervention, that consists of the removal of the whole breast, including the skin, the major and minor pectoralis muscles and of the homolateral axillary lymph nodes, found its reasoning in the basic concept that presumed a local-regional diffusion of neoplasia initially, through lymphatics, and only systemically later; a “radical” intervention was therefore necessary in such context. Other authors who head towards a less mutilating surgery demonstrated that, by saving the major pectoralis muscle (Patey’s modified radical mastectomy), or by saving both pectoralis muscles (Madden’s modified radical mastectomy), the same results could be obtained with lower morbidity, in terms of long-term survival and incidence of local relapse, respect to Halsted’s mastectomy [6,7].

The aim of conservative mastectomy is to improve patient’s quality of life avoiding aesthetic mutilation of the breast and thus reducing the negative psychologic impact, without increasing the risk of local relapse. Over the years conservative mastectomy techniques have had remarkable progress, assuming to perform a radical oncologic operation, thus with a curative purpose, saving all of the possible tissue by preserving skin and/or nipple–areola complex, maintaining the structure of breast as much as possible similar to the original. In 1980 Toth and Lappert [8] introduced the skin-sparing mastectomy; the operation consists in the removal of the whole mammary gland, saving the skin, including also removal of the nipple–areola complex. Afterward Simmond introduced the areola-sparing technique [9], which consists in the removal of the nipple only, preserving skin and areola, because it was thought that eventual metastatic cells could be present in galactophorous ducts that terminate in the nipple and not in the areola. The skin-reducing mastectomy is an immediate reconstruction with prosthetic implants for patients affected by mammary neoplasia. It can be performed in case breast volume is of medium-large dimensions and with a good degree of ptosis: the removal of skin tissue excess will allow an immediate reconstruction with prosthetic implants that will be, as much as possible, similar to the original breast in shape and dimensions.

### 1.3. The Subcutaneous Mastectomy with Preservation of the Nipple–Areola Complex (Nipple-Sparing Mastectomy)

The surgery involves the excision of the entire mammary gland preserving the skin and the nipple–areola complex and is a prerequisite for immediate reconstruction with implants. One of the first studies on the nipple-sparing mastectomy (NSM) was conducted at the Mayo Clinic on 639 women [10]. The findings published by Hartmann in 1999 showed a reduction in breast cancer incidence exceeding 90% independent of the conservation of the nipple–areola complex. The NSM mastectomy is indicated especially in the presence of medium and small sized breasts, in the treatment of large cistoadenomi phyllodes, of voluminous or recurring non-epithelial neoplasms, in recurrences after conservative treatment; another indication, far more discretionary, is that of multifocal ductal carcinomas in situ and for small infiltrating multicentric carcinomas. The indication for surgery must also consider the local situation, in terms of distance of the neoplasm from the nipple–areola complex, of gland and tumor size ratio; relative contraindications include age, heavy smokers patients, diabetic, with voluminous breasts or with ptosis, presence of autoimmune diseases with impairment of microcirculation, previous recent peri-retroareolar surgery, prior radiotherapy or prevision of adjuvant radiotherapy. Absolute contraindications are clinical and instrumental evidence of infiltration of the nipple–areola complex, Paget’s disease, presence of nipple hematic secretion with positive cytology, evident skin involvement (T4a), inflammatory carcinoma. In addition, this type of surgery, performed bilaterally, is taken into consideration as a prophylactic measure in patients at high familial risk with positive findings at genetic testing for the BRCA 1 and 2 [11,12]. The present study is a retrospective analysis aimed to evaluate the long-term outcomes of the nipple sparing mastectomy. We considered the complications, subdividing them in early and late, local and regional relapse and possible disease progression.

## 2. Materials and Methods

### 2.1. Patients’ Recruitment and Study Design

The present study is based on a retrospective analysis of NSM performed at our Institute from 2002 to 2017. We observed 1017 patients with a median age of 47 years old (range 22–76), as candidates of NSM for a follow-up time ranging from 18 to 60 months. Of these, 112 have undergone skin-sparing mastectomy and 11 areola-sparing mastectomies, in which the extemporaneous histological examination of the retroareolar tissue resulted positive for neoplastic infiltration. The data used was acquired by the Institute’s database, and includes population’s characteristics such as age, familiarity, smoking, body mass index (BMI), eventual adjuvant and neoadjuvant therapy and tumor’s features, such as tumor histotype, the stage, grading (G), hormonal status such as Estrogen Receptors (ER) and Progesterone Receptors (PgR), HER2 and Ki67 status, definitive histologic diagnosis, and relapses. Patient reconstructive outcomes, including nipple–areola complex necrosis and infection among other variables, were collected. We also analyzed the recurrence of the breast cancer in the area where the cancer was originally diagnosed (local relapse); in the lymph nodes in the armpit or collarbone area near where the cancer was originally diagnosed (regional relapse) and another part of the body such as the lungs, bones or brain (distant relapse). The complications detected were considered as “early” within 30 days later the NSM or “late” after this time. Only patients underwent NSM, with long-term follow-up (greater than 10 months) were included in the final analysis. Hospitalization after the surgery has had varying lengths depending on the cases, for a minimum of 48 h of clinical observation.

### 2.2. Surgical Technique

There are several possibilities, the skin incision should be chosen case by case in order to ensure oncological radicality and the vascular circulation of the areolar complex. The radial “italic S” incision, the periareolar or transareolar incision or the incision in correspondence to the inframammary fold, represent a good compromise in most cases. The dissection is performed in correspondence of the superficial sheet, fascia superficialis, the strip is lifted and the subcutaneous tissue is dissected with an electrical scalpel. The thickness of the subcutaneous tissue must be uniform for the whole extension of the strip. A residual thickness of 2–3 mm at the level of the nipple–areola complex, in a high percentage of cases, allows the complete excision of the ducts, while maintaining a good blood supply. However, the distance between the nipple–areola complex and the lesion was not less than 2 cm. An extemporaneous histological examination of the retro areolar ducts was carried out for each surgery, proceeding to the removal of the nipple–areola complex in the cases resulting positive for obvious contraindication to NSM. The isolation of the mammary gland must be carried out until the visualization of the retromammary muscle plan. Upon reaching the glandular extremities, we proceed with the electrical scalpel to unsticking the gland from the pectoral fascia; this is removed if the surgery is performed for small infiltrating carcinomas. After incising the skin and subcutaneous tissue the axillary fascia is reached, which will be opened along the margin of the major pectoralis muscle, allowing access to the axillary cavity. It is then possible to start searching for the sentinel lymph node through the probe for radio-guided surgery. Once identified the point of maximum radioactivity, the sentinel lymph node is removed and sent to the pathologist for an extemporaneous histological examination that will provide information on the eventual need to perform an axillary lymphadenectomy.

### 2.3. Immediate Reconstruction

All patients were subject to immediate reconstruction with tissue expanders 30% of the volume pre-filled or definitive implants. In brief, after having the hemostasis under control, we proceed to the placement of a breast implant, preferably in the retropectoral or prepectoral area, it depends on the thickness of the skin to avoid ischemic problems. The retropectoral implant For reconstruction a definitive prothesis [13] or a tissue expander [14] can be used, the decision depends on circumstances. The placement of a drainage in the pocket of the prothesis precedes the double layer suture of the wound: A first layer is performed with separated intradermal inward stitches made of reabsorbable material; the second layer with a continuous intradermal suture in monofilament, reabsorbable or non reabsorbable. Compared to modified radical mastectomies, NSM is sometimes technically more challenging and longer lasting: the skin flaps to prepare, in fact, are wider so that their isolation and manipulation requires great care, in order to prevent postoperative ischemic complications (flap necrosis).

### 2.4. Statistical Analysis

Descriptive statistics were used to describe the patient’s characteristics. The proportions are presented as numbers and percentages. Univariate and multivariate analysis of complications and prognostic factors were made using the statistical software package SPSS for Windows (SPSS Version 24, IBM Statistics; Armonck, NY, USA). The variables that were considered significantly different at a level of *p* ≤ 0.05.

## 3. Results

### 3.1. Characteristics of Tumor Relative to the Analyzed Population

From 2002 to 2017 there have been 894 NSM, the average age of the patients was 47.5 years old (range 22–76). The most histotypes represented was the infiltrating ductal carcinoma (67%) and the ductal carcinoma in situ (22.3%), followed by the infiltrating lobular carcinoma (3.9%) and the lobular carcinoma in situ (3.6%), while other forms appear in smaller percentages. The hormonal status was characterized by estrogen positivity (87.1%) and progesterone positivity (81.5%), Her2 negative (91.6%), Ki67 positive (36.1%). In the analyzed population, 215 (24%) patients underwent neoadjuvant therapy, 264 (29.5%) patient performed adjuvant therapy and while the radiotherapy was performed in 87 patients underwent lymph node dissection and resulted positive for macrometastases after lymph node dissection (Table 1).

### 3.2. Complications and Relapses

The adverse events detected were divided in early (16.8%) and late (34.2%), if present within 1 month later the NSM or later, respectively. As shown in Table 2A, the main early complication was the necrosis of the nipple–areola complex found in 6.4%, while the less detected was the skin flap necrosis (0.6%). On the other side, the capsular contracture was the most common adverse event found as “late complication” (22.2%). However, the majority of patients underwent NSM did not show early or late complications (83.2% vs. 65.8%, respectively). The local relapse rates, at the level of the nipple–areola complex (NAC) and skin was 4.9%. All 44 patients with cancer recurrence at the NAC underwent wide local excision and received a combination of systemic hormone therapy, chemotherapy and radiation therapy. Regional relapse was present in 32 patients, who reported lymph node metastasis (3.6%) and in 26 patients were detected distance metastasis (2.9%) (Table 2B).

The overall rate of locoregional relapses was 8.5% (76 patients), 8% (6 patients) of them underwent post mastectomy radiotherapy (RT). In 50% of cases the relapse was diagnosed 8 years after the diagnosis, while three relapses occurred within two years and the rest between two to five years after diagnosis.

### 3.3. Evaluation of Prognostic Risk Factors for Complications

In order to identify factors that correlate with complications, either early or later, it has been processed a univariate statistical analysis. We took into account some parameters such as, age, BMI, smoking, neoadjuvant and adjuvant chemotherapy, radiotherapy or the type of incision used, to evaluate their influence on the complications following the NSM. The italic S incision at the right upper quadrant level was the most widely used (50.3%), followed by that at the inframammary fold level (35.5%), and the radial one (14.2%). Adjuvant chemotherapy (CT) and RT were administered one month later than surgery and consequently, they were considered as prognostic factors only for late complications. The statistical analysis showed the adjuvant chemotherapy as the only predictive factor for late complications (OR (odds ratio) 1.76, *p* = 0.01), while we encountered no significant predictors for early complications (Table 3).

## 4. Discussion

The nipple–areola complex is the most important component of the identity of a female breast, the removal of which is perceived by the woman as a mutilation and for that reason it is important to always try to preserve it, so as to maintain as much as possible the original appearance of the breast itself. Necrosis and the risk of relapse at the level of the nipple–areola complex represent the specific risks of the NSM. Considering that the excision of the nipple–areola complex is experienced by the women as a mutilation, it is important to preserve it despite the risk of necrosis and recurrence. We analyzed 16 studies reporting overall survival, disease free survival, local and nipple–areola recurrence, in 1200 patients underwent NSM (Table 4). 

The mean follow-up months was 41.2, with a follow-up time ranging from 15.7 to 101 months. In all studies, including our study, the invasive ductal cancer was the most represented tumor. The average rate of local recurrence was in line with our results which detected a total local relapse rate (NAC and skin) of 4.9%. The overall complications rate (early and late) of this study is comparable with those reported in the literature [15,16,17] and the reported risk of recurrence is low [18]. According to Stolier et al., there are not terminal duct-lobular units inside the nipple in 91% of cases and only in 9% of cases the duct-lobular units are few and located at the base of the nipple [19]. This information is useful because most cancers originate from the terminal duct-lobular unit. On the other side, Rusby et al. have shown that most of the ducts (>20) emerge from some small orifices at the tip of the nipple; these ducts include a central group that narrows at the entrance of the breast parenchyma [20]. The authors [21] describe the possibility to leaving the peripheral skin margins of the nipple about 2–3 mm thick with a complete excision of the ducts in 90% of patients. However, it remains difficult to predict the integrity of the microcirculation after mastectomy and the question about the quantity of tissue of the nipple–areola complex to remove remains controversial. It is known that the attempting to removal all of retroareolar tissue exposes to a risk of necrosis whose incidence is 0.7–11% [22] but it is necessary also to minimize the risk of recurrence. In our study, the percentages of necrosis of the nipple and of the nipple–areola complex are respectfully 2.8% and 6.4%. Patients who are obese, smokers, diabetics and with breast ptosis of III and lV degree are generally not considered good candidates for NSM [23], although some studies suggest that these features do not have a significant impact on this type of complication [24]. Also, the type of incision is considered a risk factor, in particular the periareolar incision is associated with an increased risk of necrosis of the nipple–areola complex. Some studies indicate the lateral incision as the best choice to preserve the vitality of the nipple–areola complex [25]. This technique is actually the most effective when storing subcutaneous fat and keeping the structure of subdermal vascular networks. Other authors claim the usefulness of preserving the major perforating vessels, in particular the perforating artery of the second intercostal space of Stolier, recommending to maintain an adequate thickness (5 mm) of the areolar strip to avoid necrosis [26]. Generally in our institute the isolation of the mammary gland is performed with scissors and not with the electric scalpel, whose use may expose to the risk of compromising the subdermal vascular network; the artery of Stolier is usually spared, as it is not necessary to dissect it given its extra glandular course. Finally, the handling of the strips is important during surgery to avoid excessive traction so as to limit the stretching of the subdermal vascular network which may cause vascular complications such as the formation of venous microthrombi. In this study, no differences were found regarding the complication rates relative to the type of incision used. Several studies have confirmed that the risk of necrosis of the nipple–areola complex is drastically reduced when the incision does not involve the complex itself, so the lateral-radial and inframammary approaches are the best choice [27,28]. The young age (<40), the negativity of the estrogen receptors (ER), the overexpression of HER2, the tumor size, the lymph–vascular invasion, the lymph nodes involvement and the proximity of the lesion to the nipple–areola complex, the high grading are risk factors for relapses [29]. In order to reduce the probability of involvement of the nipple–areola complex, several studies affirm to check that the distance between this and the lesion is not less than 2 cm. In agreement with the literature, nipple involvement occurs in 50% of cases where the distance between the injury and the nipple–areola complex is <2 cm and in 15–20% when >2 cm [7]. Also tumor size seems an important factor, with a nipple involvement of 7–20% for tumors of <2 cm which rises up to 30% for lesions > 2 cm and 50–80% for lesions > 4 cm [30]. Therefore, in the hypothesis of proceeding with a NSM, the surgeon must consider the size of the lesion and the distance between this and the nipple–areola complex; however in cases where this distance is reduced, but there is no certainty on retroareolar infiltration in the preoperative investigations, preservation of the nipple–areola complex may be possible. The low local relapse rate (1.6%) of our study is due primarily to a careful selection of patients to submit to NSM, with special attention to the study of the lesion site using methods of imaging, such as ultrasound and magnetic resonance imaging (MRI). The cases of local relapse also show poorer prognosis, being associated with reduced long-term survival rates [31], which require close monitoring over time. The global survival rate relative to the present study is 98.8%. This is due to the good prognosis of patients selected; in fact the 75.2% of patients were at the I–IIA and IIB stage, a grading of 3 was present in 41% of the cases, more than 80% of patients had hormonal receptors that were positive, HER2 was positive only in 8% of cases and the Ki67 was high (>20%) in 36% of cases. A recent meta-analysis that analyze the complications and the oncological safety of the nipple-sparing mastectomy, found similar results in term of global survival, disease-free survival and local recurrence [32].

## 5. Conclusions

This study adds to the data already present in literature, demonstrating that the NSM is a safe procedure, characterized by a survival and relapse rates that are substantially similar to those of other more invasive surgical procedures such as skin-sparing mastectomy, modified radical mastectomy, and areola-sparing mastectomy. In conclusion, the NSM should be considered the best choice of surgery, whenever possible, because every woman has the right to treatment and survival while avoiding whenever possible the psychological trauma due to an aggressive surgery.

## Figures and Tables

**Table 1:** Representation of some characteristics of the analyzed population.

Characteristics	Patients (*n* = 894)
**Age**; years, mean (range)	47.5 (range 22–76)
**Age**; *n*, (%) (<47 vs. ≥47)	295 (33) vs. 599 (67)
**BMI**; *n*, (%) (<30 vs. ≥30)	733 (82) vs. 161 (18)
**No Smoking vs. Smoking***n*, (%)	692 (77.4) vs. 202 (22.6)
**Histology**; *n*, (%)	
Invasive ductal carcinoma	599 (67)
In situ ductal	200 (22.4)
Invasive lobular carcinoma	51 (5.7)
In situ lobular	32 (3.6)
Tubular carcinoma	5 (0.6)
Medullary carcinoma	3 (0.3)
Mucinous carcinoma	2 (0.2)
Invasive papillary carcinoma	1 (0.1)
Metaplastic carcinoma	1 (0.1)
**Molecular Marks**; *n*, (%)	
ER+	779 (87.1)
ER−	115 (12.9)
PgR+	729 (81.5)
PgR−	165 (18.5)
HER2+	71 (8)
HER2−	823 (92)
Ki67+	323 (36.1) (>20%)
Ki67−	571 (63.9) (≤20%)
**Therapies**; *n*, (%)	
CT neoadjuvant	215 (24)
CT adjuvant	264 (29.5)
RT	87 (9.7)
**Incision**; *n*, (%)	
italic “S” vs. inframammary fold	450 (50.3) vs. 317 (35.5)
radial vs. inframammary fold	127 (14.2) vs. 317 (35.5)

BMI—body mass index; ER—Estrogen Receptors; PgR—Progesterone Receptors; HER2—human epidermal growth factor receptor 2; CT—Chemotherapy; RT—Radiotherapy.

**Table:** (**A**)

Early Complications	*n*, (%)	Late Complications	*n*, (%)
Nipple–areola necrosis	57 (6.4)	Capsular contracture	199 (22.2)
Depigmentation	28 (3.1)	Prosthesis dislocation	73 (8.2)
Nipple necrosis	25 (2.8)	Prosthesis extrusion	34 (3.8)
Prothesis infection	20 (2.2)	No complications	588 (65.8)
Epidermolysis	15 (1.7)		
Skin flap necrosis	5 (0.6)		
No complications	744 (83.2)		

**Table:** (**B**)

Local Relapses	Regional Relapses	Distant Relapses
44 (4.9%)	32 (3.6%)	26 (2.9%)

**Table 3:** Statistical analysis of prognostic factors for complications.

Variables	Early (≤1 month) OR (CI 95%)	*p*	Late (≥1 month) OR (CI 95%)	*p*
Adjuvant CT ^1^	-		1.76 (1.13–2.75)	0.01 *
Neoadjuvant CT	1.25 (0.45–3.50)	0.66	1.85 (0.82–4.20)	0.14
RT ^1^	-		1.22 (0.68–2.21)	0.51
BMI (<30 vs. ≥30)	0.85 (0.55–1.33)	0.49	1.02 (0.97–1.10)	0.37
Age (<47 vs. ≥47)	0.78 (0.53–1.15)	0.22	1.14 (0.73–1.79)	0.54
Incision				
italic “S” vs. inframammary fold	1.15 (0.78–1.71)	0.48	1.34 (0.81–2.22)	0.25
radial vs. inframammary fold	1.04 (0.60–1.80)	0.88	1.31 (0.62–2.78)	0.48
Smoking	1.36 (0.91–2.03)	0.13	1.42 (0.87–2.33)	0.16

^1^ Adjuvant CT and RT were administered one month later than surgery and consequently, they were considered as prognostic factors only for late complications. OR—odds ratio; CI—confidence interval. * statistically significant.

**Table 4:** Global survival, disease-free survival, local relapse, and areola–nipple complex from meta-analysis in the literature.

Author	NSM (*n* = 1200)	Follow-up (Months)	Overall Survival (%)	Disease-Free Survival (%)	Local Recurrence (%)	NAC Recurrence (%)
Adam et al. [1]	67	36	96.2	94.1	0	0
Boneti et al. [2]	152	25.3	-	-	4.6	-
Burdge et al. [3]	39	25.3	97.4	-	10.3	0
Caruso et al. [4]	50	66	92	88	0	2
Crowe et al. [5]	83	41	98.8	95.1	0	1.2
Gerber et al. [6]	60	101	76.7	-	11.7	1.7
Jensen et al. [7]	77	60.2	100	100	0	0
Kim et al. [8]	152	60	97.1	89	2	1.3
Nava et al. [9]	58	36	98.2	94.9	1.6	0
Paepke et al. [10]	94	34	98.9	94.7	1.1	0
Poruk et al. [11]	105	25.8	96.2	92.4	1.9	0
Sacchini et al. [12]	68	24.6	98.5	95.6	2.9	0
Shi et al. [13]	35	68	94.3	82.9	5.7	2.9
Sood et al. [14]	76	15.7	98.7	91.9	7.9	1.3
Tancredi et al. [15]	55	21.7	100	92.7	0	3.6
Voltura et al. [16]	29	18	96.6	93.1	6.9	0
